# Oxidative and Antioxidative Status Expressed as OSI Index and GSH/GSSG Ratio in Children with Bone Tumors after Anticancer Therapy Completion

**DOI:** 10.3390/jcm11061663

**Published:** 2022-03-17

**Authors:** Joanna Gajewska, Magdalena Chełchowska, Magdalena Rychłowska-Pruszyńska, Teresa Klepacka, Jadwiga Ambroszkiewicz

**Affiliations:** 1 Department of Screening Tests and Metabolic Diagnostics, Institute of Mother and Child, Kasprzaka 17a, 01-211 Warsaw, Poland; magdalena.chelchowska@imid.med.pl (M.C.); jadwiga.ambroszkiewicz@imid.med.pl (J.A.); 2 Department of Oncology and Surgical Oncology for Children and Youth, Institute of Mother and Child, Kasprzaka 17a, 01-211 Warsaw, Poland; magdalena.rychlowska@imid.med.pl; 3 Pathomorphology Department, Institute of Mother and Child, Kasprzaka 17a, 01-211 Warsaw, Poland; teresa.klepacka@imid.med.pl

**Keywords:** oxidative stress, glutathione, osteosarcoma, Ewing’s sarcoma

## Abstract

Aims. There are no data on the redox status of children with bone tumors in complete disease remission. Therefore, the presented study examined the reduced/oxidized glutathione (GSH/GSSG) ratio, total oxidant capacity (TOC) and total antioxidant capacity (TAC) values as well as the oxidative stress index (OSI) for assessing alterations in the oxidant/antioxidant balance in 35 children with osteosarcoma or Ewing’s sarcoma after anticancer therapy completion (median 14 months) compared with a control group. Methods. GSH, GSSG, TOC, TAC concentrations and bone alkaline phosphatase (BALP) activity were evaluated by immunoenzymatic (ELISA) and enzymatic methods. Results. We found no differences in serum BALP activity between all survivors with bone tumors and the control group. Patients with osteosarcoma after anticancer therapy completion had significantly higher values of TAC, GSH and the GSH/GSSG ratio as well as GSSG than healthy subjects. In patients with Ewing’s sarcoma, we found significantly higher values of TOC concentration compared with healthy children. In addition, survivors with Ewing’s sarcoma had higher TOC concentrations and OSI index values (*p* < 0.01), but a lower GSH/GSSG ratio (*p* < 0.05) than survivors with osteosarcoma. A positive correlation between TOC and the post-therapy period was observed in survivors. Conclusions. We found that in survivors with bone tumors, a disturbed balance between prooxidants and antioxidants persists after the completion of anticancer treatment. Moreover, an increased TOC value together with the post-therapy period may suggest increasing oxidative processes in survivors with bone tumors after treatment. Further observations will allow assessment of the relationship between the oxidant/antioxidant status and the predisposition of survivors to bone neoplastic disease recurrence.

## 1. Introduction

The redox balance has been suggested to be an important factor for cancer initiation and progression [1]. Cancer cells exhibit elevated oxidative stress, which may be a consequence of the high metabolic rate and/or the activation of ROS-coupled signaling pathways. In addition, antioxidant levels are often elevated in cancer cells and tumors, leading to cellular adaptation and redox rebalance. There is a frequent association with the progression of neoplastic disease and the development of drug resistance [2].

One of the detectable markers of the antioxidant defense system in the plasma is glutathione (γ-glutamyl-cysteinyl-glycine), a peptide with various functions such as detoxification, antioxidant defense and regulation of cellular proliferation [3]. Glutathione includes two forms: a reduced form (GSH) and an oxidized form (GSSG). The GSH/GSSG ratio tends to decrease in severe oxidative stress and the accumulation of GSSG, leading to a decreased body defense against free radicals. The increased production of oxidizing species or a significant decrease in the effectiveness of antioxidant defense may play an important role in the etiology of cancers such as lung, breast, primary bone and soft tissue sarcoma [4,5,6].

Due to metabolic and signaling aberrations, cancer cells exhibit elevated ROS levels, and this is balanced by an increased antioxidant capacity [7]. Many cancer types, including liver, lung, breast and colon cancers, show elevated GSH levels in comparison with normal tissues, and this makes the neoplastic tissues more resistant to chemotherapy [8,9]. The level of GSH in some tumor cells is associated with higher activities of GSH-related enzymes, such as γ-glutamylcyclotransferase (GGCT) and γ-glutamyl-transpeptidase (GGT), and a higher expression of GSH-transporting export pumps [10,11]. Moreover, GSH turnover or utilization are under the transcriptional control of tumorigenic pathways, mainly the nuclear factor erythroid 2-related factor 2 (Nrf2) signaling, which influences the antioxidant response [12]. High GSH/GSSG ratios in cancer cells can also be explained by an increased flux through the pentose phosphate pathway (PPP), which produces nicotinamide adenine dinucleotide phosphate (NADPH). As a result, these cells significantly accumulate GSH and PPP-related metabolites [13,14].

Osteosarcoma (OS) is the most frequent malignant bone neoplasm, followed by chondrosarcoma and Ewing’s sarcoma (ES) [15]. The peak incidence of OS is at age 10–30 years. Among the most affected are the femur, tibia and humerus. Eighty percent of all metastases arise in the lungs and exhibit resistance to conventional chemotherapy [16]. The five-year survival rate for OS patients with metastases is 20% compared with 65% for patients with localized disease [17]. Ewing’s sarcoma, belonging to the Ewing’s sarcoma family of tumors (ESFT), is an aggressive childhood cancer likely originating from mesenchymal stem cells or osteo-chondrogenic progenitors [18].

ES is a rapidly metastasizing cancer; hence, about 25% of cases are metastatic at initial diagnosis. EWS gene rearrangement on chromosome 22q12 was observed to underlie the development of Ewing’s sarcoma. Despite the intensification of treatment strategies (chemotherapy and sometimes, additionally, radiotherapy), the ten-year survival of those with metastatic disease is below 30% due to disease progression [19]. Radiotherapy can further increase the production of reactive oxygen species in patients with ES. Accumulation of ROS plays a role in the dysregulation of the antioxidant system contributing to increased oxidative stress and bone loss by enhancing the bone resorption process [20].

Several studies have reported that increased oxidative stress also has a negative impact on bone formation by modulating the differentiation and survival of osteoblasts [20,21]. The increased ROS leads to cellular damage, such as increased osteogenic cell apoptosis and decreased osteoblast number. However, reactive oxygen species are also produced during physiological process-bone remodeling that requires the generation of new osteoblasts from mesenchymal stem cells (MSCs). The differentiating MSCs may activate the antioxidant defense system to prevent ROS accumulation [22].

Enzymatic and non-enzymatic antioxidants as well as their synergistic effects can be evaluated by serum total antioxidant capacity (TAC), whereas the intensity of oxidation processes can be measured by total oxidant capacity (TOC). The estimation of a concentration for each antioxidant separately, as well as the total free radical scavenging capacity, may also be useful in the assessment of the oxidant/antioxidant balance. In adults, in both serum and tissue samples from breast cancer patients, total oxidative status (TOS) was significantly increased in comparison with a control group and noncancerous tissues of breast cancer patients [23]. Increased TOS values in other types of cancer such as thyroid and colorectal cancers were also observed [24,25]. During anticancer therapy, a decrease of TAC was found, but in cancer survivors, this parameter was not different from that in the control group [26,27]. In pediatric patients with tumors, including osteosarcoma and Ewing’s sarcoma, various levels of the GSH/GSSG ratio in serum were obtained before treatment [28]. However, there are no data on the oxidative and antioxidative status of children with bone tumors in complete disease remission. Therefore, the presented study examined the GSH/GSSG ratio, TOC and TAC values as well the oxidative stress index (OSI), which express the TOC/TAC ratio as tools for assessing alterations in the oxidant/antioxidant balance in the serum of patients with osteosarcoma and Ewing’s sarcoma after anticancer therapy completion in comparison with a control group.

## 2. Materials and Methods

### 2.1. Patients

**Figure 1:**
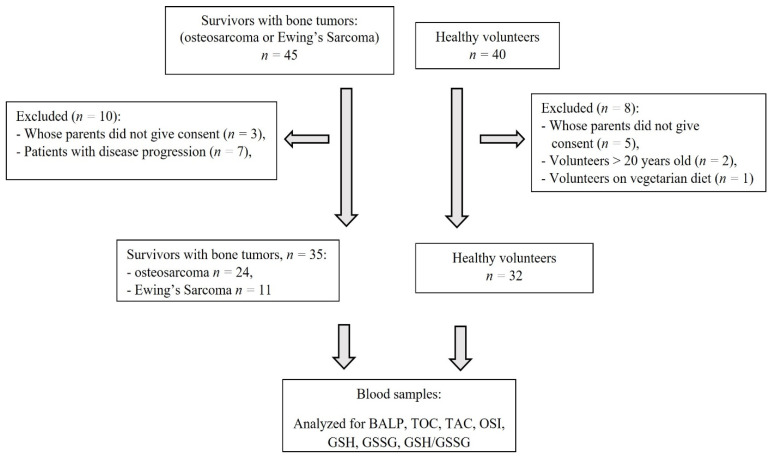
Flowchart of the study. GSH—reduced glutathione, GSSG—oxidized glutathione, TOC—total oxidant capacity, TAC—total antioxidant capacity, OSI—oxidative stress index (TOC/TAC), BALP—bone alkaline phosphatase.

The studied group consisted of 35 Caucasian children (20 male and 15 female) aged 9–20 years (median: 16 years) with osteosarcoma or Ewing’s sarcoma, treated at the Institute of Mother and Child in Warsaw. Bone tumor diagnosis, established by clinical and radiological findings, was confirmed histologically at the surgical biopsy of the primary tumor. The reference group consisted of 32 healthy children (18 male, 14 female) of the same age and race, without: (a) diseases affecting growth rate and bone metabolism, (b) other chronic medical conditions or (c) intake of medications that could affect growth, pubertal development, nutritional or dietary status (Figure 1).

The clinical characteristics of the patients are summarized in Table 1.

Survivors with osteosarcoma or Ewing sarcoma were in complete remission at the time of the study. The evaluation of oxidative and antioxidative status in these patients was performed at 14 months (7.5–17.0 months) after the completion of antitumor treatment. The patients who had received standard treatment which consisted of neoadjuvant (preoperative) chemotherapy followed by tumor resection and adjuvant (postoperative) chemotherapy were included in this study. In the study group, there were 24 patients with OS and 11 patients with ES. In most patients, the primary tumor was localized in the femur.

Patients with osteosarcoma received preoperative chemotherapy (according to EURAMOS) composed of high-dose methotrexate, cisplatin and doxorubicin. The histopathological examination revealed osteoblastic osteosarcoma in 15, chondroblastic in 3, telangiectatic in 1 and other forms of OS in 5 patients. Twenty-three patients had a good response to initial chemotherapy (>90% overall tumor necrosis), and in twelve, necrosis was <90%. Histopathological grades of GI, GI/GII, GII, GII/GIII and GIII were found in two, two, six, eight and six patients with OS, respectively. Further adjuvant chemotherapy was carried out in patients with tumor necrosis of >90% who received the same type of chemotherapy, whereas patients with poor response to treatment and with tumor necrosis of <90% of overall tumor mass received intensified adjuvant chemotherapy according to the MAPIE protocol (varying combinations of methotrexate, cisplatin, doxorubicin, ifosfamide and etoposide).

The group of patients with Ewing’s sarcoma received chemotherapy according to the Euro-Ewing 99 protocol (neoadjuvant–according to VIDE: vincristine, ifosfamide, doxorubicin, etoposide and adjuvant, according to VAI or VAC: vincristine, actinomycin-D, ifosfamide or cyclophosphamide). Among the 11 patients with ES, 6 patients (5 boys/1 girl) underwent radiotherapy.

The average duration of treatment in the whole group of patients was 12.5 ± 4.5 months. All patients were monitored at the Institute of Mother and Child after treatment cessation.

The study was performed in accordance with the Helsinki Declaration for Human Research, and the study protocol was approved by the Ethics Committee of the Institute of Mother and Child in Warsaw, Poland (approval no. 03/09). Informed consent was obtained from the study participants or their parents.

### 2.2. Methods

The material for biochemical analysis was venous blood collected from the patients and healthy controls in the morning after an overnight fast. Serum or EDTA plasma obtained after centrifuging the blood (2500× *g*, 4 °C, 10 min) was stored in small portions until analysis was performed (−70 °C, less than 2 months).

Total oxidant capacity and total antioxidant capacity values were measured in plasma by colorimetric assay based on the enzymatic reaction of peroxides and peroxidases according to Tatzber et al. [29] (Omnignostica Forschungs GmbH, Hoflein/Danube, Austria). Oxygen produced by this reaction oxidizes the chromogenic substrate tetramethylbenzidine (TMB). By adding sulfuric acid, the reaction cascade is stopped and the color of the mixture changes from blue to yellow and can be detected at 450 nm. The concentrations of the standards are given in H_2_O_2_ equivalents (mmol/L). Plasma peroxide levels were calculated as the difference in the absorbance readings relating to the hydrogen peroxide standard curve. Antioxidants inhibit this reaction and can be detected analogously on the basis of the indirect proportionality of this inhibition reaction. The analytical sensitivity of TOC was 0.06 mmol/L, and the intra- and inter-assay coefficients of variation (CV) were 4.9% and 7.3%, respectively. The sensitivity of TAC was 0.08 mmol/L, and the intra- and inter-assay CV were 5.0% and 6.9%, respectively. The oxidative stress index (OSI) was calculated from the measured values of TOC and TAC using the TOC/TAC ratio.

The reduced and oxidized glutathione were measured in plasma using the Human GSH ELISA kit and the Human GSSG ELISA kit (SunRed Biotechnology Company, Shanghai, China), respectively. The kits use a double-antibody sandwich enzyme-linked immunosorbent assay to determine the level of GSH or GSSG in the samples. These are the assays in a microtiter strip format utilizing a human monoclonal anti-GSH (-GSSG) antibody coated on the strip to capture GSH (GSSG) from the sample in the presence of the GSH (GSSG) antibodies labeled with biotin and combined with streptavidin-HRP to form an immune complex. After adding chromogen solutions, the color of the liquid changes to blue, becoming yellow after the reaction has stopped. Absorbance was measured at 450 nm. The analytical sensitivity of GSH was 0.118 µmol/L, and the intra- and inter-assay coefficients of variation (CVs) were lower than 10%. The analytical sensitivity of GSSG was 0.045 µmol/L, and the intra- and inter-assay CVs were lower than 8% and 11%, respectively. The GSH/GSSG ratio, which is considered an index of the cell’s redox, was calculated.

Serum bone alkaline phosphatase (BALP) activity was determined using the MicroVue BAP kit from Quidel (San Diego, CA, USA). This immunoassay provides a quantitative measure of bone-specific alkaline phosphatase activity in serum as an indicator of osteoblastic activity. MicroVue BAP is an assay in a microtiter strip format utilizing a monoclonal anti-BALP antibody coated on the strip to capture BALP in the sample. The enzyme activity of the captured BALP is detected with p-Nitrophenyl phosphate (pNPP) substrate at 405 nm. The limit of detection for BALP was 0.7 U/L. The intra- and inter-assay CVs for BALP were below 6% and 8%, respectively.

### 2.3. Statistical Analysis

Statistical analysis was performed using STATISTICA 10.0 (StatSoft Inc., Tulsa, OK, USA). The Kolmogorov–Smirnov test was used to assess the normality of variable distribution. Results were presented as mean values and standard deviation (SD) for symmetrically distributed data or as median values and interquartile range (IQR) for asymmetrically distributed data. Group differences were assessed using the student’s *t*-test for normally distributed data and the non-parametric Mann–Whitney U test for non-normally distributed variables. First, differences in biochemical parameters of the whole group of bone tumor survivors and healthy children were assessed. For the next analysis, patients were divided into two subgroups stratified by type of tumor using patients with OS (*n* = 24) and patients with Ewing’s sarcoma (*n* = 11). The patient subgroups were also compared with the healthy subjects. The studied parameters were analyzed separately in the groups of girls and boys with bone tumors. Spearman correlations between biochemical parameters as well as the post-therapy periods were calculated. Differences were regarded as statistically significant at *p* < 0.05.

## 3. Results

Bone tumor survivors and healthy children did not differ in terms of age (Table 2).

Serum TAC concentration was higher (*p* < 0.01) in bone tumor survivors than in healthy children, but no significant differences in serum TOC concentration as well as the OSI index were observed between these groups. Serum GSH concentration was higher by about 60% (*p* < 0.001), but GSSG concentration was only slightly higher by 15% (*p* = 0.054) in bone tumor survivors than in the healthy group. A significantly higher value of the GSH/GSSH ratio (*p* = 0.001) was also observed in the patients group. There were no differences in serum BALP activity between survivors and the control group.

The differences in the biochemical parameters between survivors with osteosarcoma and Ewing’s sarcoma are presented in Table 3.

Patients in both studied subgroups did not differ in terms of age and the post-therapy period. Survivors with Ewing’s sarcoma had higher TOC concentration and the OSI index value (*p* < 0.01), but a lower GSH/GSSG ratio (*p* < 0.05) than survivors with osteosarcoma. Serum BALP activity, TAC and GSH levels were slightly lower in Ewing’s sarcoma survivors than in osteosarcoma survivors, but this was not statistically significant. If the studied parameters were analyzed separately in the groups of girls and boys with bone tumors, some sex-dependent differences were observed. Boys with Ewing’s sarcoma had a significantly higher TOC concentration (0.264 ± 0.040 vs. 0.167 ± 0.068 mmol/L, *p* = 0.002) and OSI index value (0.188 (0.177–0.197) vs. 0. 0.104 (0.057–0.140), *p* = 0.010), but lower value of GSH (17.4 (13.4–19.3) vs. 26.5 (16.0–53.4) µmol/L, *p* < 0.007) than boys with osteosarcoma. Similar tendencies were observed when analyzing the studied parameters in both groups of girls with osteosarcoma and Ewing’s sarcoma, but these differences were not statistically significant.

In patients with Ewing’s sarcoma after the completion of anticancer therapy, we found a higher value of TOC concentration (*p* = 0.025), but similar TAC concentration (*p* = 0.328) and GSH/GSSG ratio (*p* = 0.151) as compared with healthy children.

Patients with osteosarcoma after completion of anticancer therapy had significantly higher values of TAC, GSH and the GSH/GSSG ratio (*p* < 0.001) as well as GSSG concentrations (*p* = 0.049) than healthy subjects. No differences in serum TOC concentration (*p* = 0.590) and the TOC/TAC ratio (*p* = 0.088) between these survivors and the control group were found.

Positive correlations between TAC and GSH (*p* = 0.026) and the GSH/GSSG ratio (*p* = 0.003) were found in healthy children (Table 4). There were no significant correlations between TOC and OSI and glutathione in these subjects.

In bone tumor survivors, positive correlations between TAC and GSH (*p* = 0.023) and GSSG (*p* = 0.014) were found (Table 5). There were no significant correlations between TOC and OSI and glutathione in these subjects.

**Figure 2:**
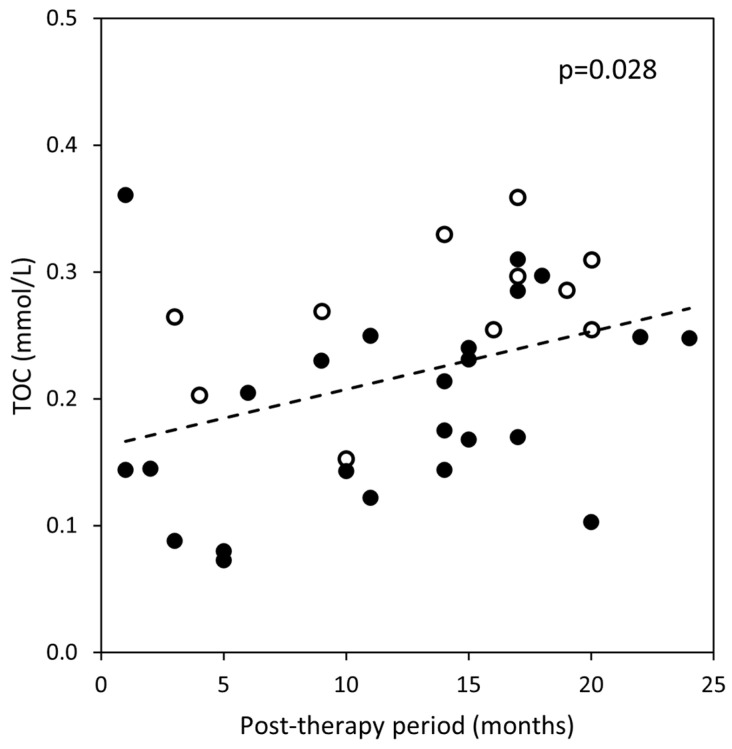
Spearman’s correlation between serum concentrations of TOC (total oxidant capacity) and the post-therapy period in bone tumor survivors (○ with osteosarcoma; ● with Ewing’s sarcoma).

However, in bone tumor survivors, a positive correlation between TOC and the post-therapy period (r = 0.371, *p* = 0.028) was found (Figure 2). There were no significant correlations between the post-therapy period and TAC (r = −0.198, *p* = 0.255) and OSI (r = 0.309, *p* = 0.071) in bone tumor survivors.

## 4. Discussion

Oxidative stress has long been implicated in cancer development and progression. ROS production is significantly increased in cancer resulting in an accumulation of oxidized molecules and altered metabolism cells because of genetic mutations and mitochondrial dysfunction [30]. However, little is still known about the oxidation/antioxidant status, its dynamics and association with clinical outcomes of patients with cancer in the post-therapy period.

In our study, all of the survivors with osteosarcoma or Ewing sarcoma were in complete remission at the time of the study. We found similar values of BALP activity in these patients and healthy children without diseases affecting growth rate and bone metabolism. In the diagnostic process, serum BALP has been shown to be associated with bone metastases of prostate cancer [31]. Levine et al. [32] found significant amounts of this de novo synthesized enzyme in human osteosarcoma cell cultures, especially in osteoblastic sarcoma. It is known that BALP activity is associated not only with the osteogenic potential of a tumor, but also with tumor cell aggressiveness and metastasis [33,34]. According to Rychłowska-Pruszyńska et al. [35], higher values of BALP were related to disease progression disease and unfavorable prognosis. These authors found three-fold higher BALP activity in osteosarcoma patients with metastases compared with patients with localized disease at the time of biopsy. After postoperative therapy in patients with disease remission, BALP activity was significantly lower than in patients with progression/metastases. In the presented study, no symptoms of bone tumor and unchanged BALP activity were observed in the studied patients after the end of treatment.

Analysis of redox status in survivors with bone tumors after the end of clinical treatment (median 14 months) demonstrated enhanced antioxidant defense, probably associated with an increased level of GSH in serum and an increased ratio of GSH to GSSG. Moreover, we observed positive associations between TAC and the reduced form of glutathione in both the control and the survivor groups.

Wu et al. [25] showed that total oxidant status and OSI levels increased significantly and the level of total antioxidant status significantly decreased in patients with colorectal cancer compared with a healthy control group. According to other authors, TAC also decreased during the treatment of acute lymphoblastic leukemia (ALL) and prostate cancer due to increased oxidative stress [26,27]. Chevion et al. [36] also observed that plasma TAC in patients with ALL was reduced during total body irradiation, but after four months, was higher by about 20% than before treatment. Dincer et al. [27] observed that serum TAC in ALL survivors was not different from those in the control group. Children with ALL were in complete hematologic remission at the time of this study. Moreover, all of the cases had no leukemia symptoms and did not receive anticancer therapy within the previous year. The authors suggested that TAC levels may be restored after the end of anticancer treatment.

In contrast to Dincer et al. [27], we observed higher TAC values after the end of treatment in the entire group of children with bone tumors, but by analyzing the subgroups, we found differences in oxidant/antioxidant status between patients with osteosarcoma and Ewing’s sarcoma. It seems that the different results obtained may depend on the type of tumor. Scibior et al. [37] found lower levels of glutathione in patients with gastric cancer and higher levels in patients with colorectal cancer liver metastases. These authors suggested that changes in glutathione metabolism are associated with different types of gastrointestinal tract tumors.

In our study, an increase in TAC and GSH levels as well the GSH/GSSG ratio was observed more often in survivors with OS than ES. Patients with Ewing’s sarcoma, compared with patients with OS, showed an increased oxidative potential OSI, which suggests more intensified oxidative processes in these survivors. In addition, a lower ratio of GSH to GSSG in patients with Ewing’s sarcoma compared with OS patients was found. Lower levels of GSH + GSSG were observed before and after surgery in breast cancer patients by other authors [38]. Grunwell et al. [39] found an increase in GSSG and a more oxidized redox state of GSH/GSSG in the serum of critically ill children aged 0–18 years (sepsis, asthma, solid tumors) compared with that of healthy children. The authors proposed that GSSG and the GSH/GSSG ratio may serve as potential biomarkers of oxidative stress.

Data on GSH focuses mainly on its intracellular contributions, and much less on GSH present in circulation. Most cells cannot readily import GSH, therefore, this peptide is catabolized into its precursor amino acids by gamma-glutamyl transferases. The import of amino acids derived from GSH breakdown may drive intracellular GSH synthesis as well as a number of metabolic pathways. It is speculated that GSH may act as an amino acid storage mechanism in the body, where GSH synthesized by specific tissues can be exported into the circulation. The glutathione pool increases in distal tissues, and then, as a result of catabolic processes to amino acids, it would intensify metabolic processes. [40].

Zhong et al. [41] observed alterations of intracellular metabolome in osteosarcoma stem cells using liquid chromatography–tandem mass spectrometry. According to these authors, the metabolomics data indicated that both the citrate cycle (TCA) and glutathione metabolism were downregulated (elevation of GSSG levels) in human osteosarcoma cell line as a result of a decline in mitochondrial metabolism. However, Wang et al. [42] found that osteosarcoma stem-like/progenitor cells adapted to gain a survival advantage by enhanced GSH synthesis due to the maintenance of resistance to endoplasmic reticulum stress-induced apoptosis. γ-Glutamylcyclotransferase in the γ-glutamyl cycle may play an important role in regulating the synthesis of glutathione by limiting the availability of γ-glutamylcysteine (γ-Glu-Cys) [43]. GGCT might also have a cell protective role through an antioxidant effect by glutathione salvage. GGCT upregulation has been reported in many cancers [44,45]. Uejima et al. [45] also examined the expression of GGCT mRNA in 40 surgical specimens of osteosarcoma compared with normal human osteoblasts as a control. Moreover, GGCT depletion inhibited the growth of cancer cell lines in vitro, including osteosarcoma, colorectal cancer and ovarian cancer cells [45,46,47]. Therefore, it is suggested that metabolic adaptation to oxidative stress would be a pro-survival phenotype, hence, antioxidant response such as altered glutathione metabolism has been found in metastatic osteosarcoma [48]. The GSH metabolic pathway was found to be significantly altered in highly metastatic OS cells compared with low metastatic counterparts. In addition, other metabolic pathways were found to be altered in highly metastatic OS cells, including arginine, inositol and lipid metabolic pathways [49].

Scotlandi et al. [50] using gene-expression microarray analysis showed that the GSH metabolism pathway is also one of the most significantly altered pathways in Ewing’s sarcoma family of tumors. It was shown that ESFT cell lines express γ-glutamyl transpeptidase, an enzyme regulating GSH homeostasis [51]. Moreover, ESFT cell lines had low superoxide dismutase (SOD) and glutathione peroxidase (GPX) activities and low GSH/GSSG ratios, indicating mild to severe oxidative stress under routine culture conditions [52]. In our study, survivors with Ewing’s sarcoma had higher TOC values with unchanged TAC values in comparison with controls as well as survivors with OS. This suggests greater intensification of oxidative processes and the elevation of ROS production in patients with ES after the end of anticancer treatment. The influence of radiotherapy on the increased oxidative stress cannot be ruled out in survivors with ES, especially in the group of boys, where we observed higher OSI index values and lower GSH concentrations after treatment. It is known that ionizing radiation (IR) generates highly reactive molecules that cause cellular damage [53]. Moreover, IR upregulates function of the mitochondrial electron transport chain intensifying mitochondrial ROS production [54]. However, it is known that ESFTs are childhood cancers characterized by aggressive behavior and a propensity for relapse. Transcription factor SOX6 (SRY-Box Transcription factor 6) is highly expressed in Ewing’s sarcoma cells that display a highly proliferative phenotype. Marchetto et al. [55] demonstrated that SOX6 stimulates the expression of Thioredoxin Interacting Protein (TXNIP), which is an inhibitor of the thioredoxin antioxidant system. These authors suggest that the SOX6-induced TXNIP expression may contribute to elevated intracellular and mitochondrial levels of oxidative stress.

The specific functions of different ROSs in the various stages of metastasis are still not well defined, but increased ROS levels are frequently associated with the activation of the epithelial-to-mesenchymal transition, cell motility and metastasis [56,57]. In our study, we found that the oxidative processes measured by TOC concentration may be more intensified over time in survivors with bone tumors after treatment. It is known that moderate ROS levels can support proliferation by activating signaling pathways that can contribute to tumor growth, but excessive ROS accumulation results in severe damage of many molecules, causing cell death. In addition, GSH may also play a role in cancer progression [9]. It is a cell protective role through an antioxidant effect and it is important in removing and detoxifying carcinogens or drugs, affecting the survival of cancer cells. Moreover, elevated GSH and other common antioxidants, such as N-acetyl cysteine and the vitamin E analogue trolox, may promote metastasis [58]. Due to the dual roles of ROS as well as GSH in cancer initiation and progression, further monitoring of survivors with bone tumors in terms of oxidant/antioxidant status as well as symptoms of relapse is necessary.

The possibility of using antioxidants in cancer therapy has been intensively studied for many years. The results of these studies are still inconclusive, as some antioxidants can reduce the efficacy of chemo- and radiotherapy [53]. In addition, moderate intake of antioxidants could be beneficial, while excess antioxidant intake may have an adverse health effect. Therefore, it is advisable to monitor antioxidant intake and patient blood levels during supplementation. However, various cancer types, different courses of chemotherapy, radiotherapy and multiple antioxidants still cause difficulties in making decisions regarding antioxidant supplementation during cancer management [2].

The present study had several limitations. First, we had a relatively small number of participants owing to the rarity of malignant bone tumors, the mortality rate and limitations resulting from the occurrence of these tumors mainly in adolescence. However, the study group was homogeneous in terms of developmental period and was well characterized clinically. The next limitation of this study was its cross-sectional nature and the absence of a prospective longitudinal analysis that is needed to examine the relationship between redox balance and clinical outcomes in these subjects. Nevertheless, it was the first study to investigate the oxidant/antioxidant status of children with osteosarcoma or Ewing’s sarcoma shortly after the completion of anticancer therapy. We are planning to conduct observations of the studied patients and repeat biochemical analyses in the future. Finally, enzymatic (glutathione-linked enzymes) and other non-enzymatic antioxidants as well as transcriptional factors related to the synthesis of antioxidant enzymes, such as Nrf2, might also be useful in evaluating total antioxidative capacity in survivors with bone tumors. This will be the subject of further research.

## 5. Conclusions

In survivors with bone tumors, a disturbed balance between prooxidants and antioxidants persists after the completion of anticancer treatment. In survivors with osteosarcoma, this appears to be related to the up-regulation of the GSH antioxidant system, but in survivors with Ewing’s sarcoma, with increased oxidative processes. In addition, the elevated TOC value along with the post-therapy period may suggest an intensification of oxidative processes over time in survivors with bone tumors after treatment. Further observations will allow for assessment of the relationship between the oxidant/antioxidant status and the predisposition of survivors to bone neoplastic disease recurrence.

## Figures and Tables

**Table 1:** Clinical characteristics of patients with malignant bone tumors.

		Number of Patients	Percent of Patients
Gender	Male	20	57%
	Female	15	43%
Type of tumor (histological subtype)	Osteosarcoma	24	69%
	osteoblastic	15	62.50%
	chondroblastic	3	12.50%
	teleangiectatic	1	4%
	mixed	5	21%
	Ewing’s sarcoma	11	31%
Localization of primary tumor	Femur	19	54.50%
	Humerus	6	17%
	Tibia	4	11.50%
	other	6	17%
Metastases	With metastases	21	60%
	Without metastases	14	40%
Histological response	>90% tumor necrosis	23	66%
	<90% tumor necrosis	12	34%
Type of surgery	Resection	30	86%
	Amputation	5	14%

**Table 2:** Differences in biochemical parameters between bone tumor survivors (osteosarcoma, Ewing’s sarcoma) and healthy subjects.

	Bone Tumor Survivors *n* = 35	Healthy Children *n* = 32	*p*
Age (years)	15.7 ± 3.2	14.0 ± 5.6	0.147
Girls/Boys (%)	42.9/57.1	43.7/56.3	0.580
Post-therapy period (months)	14.0 (7.5–17.0)	not applicable	not applicable
BALP (U/L)	78.8 (41.2–127.3)	98.7 (65.0–119.3)	0.467
TAC (mmol/L)	1.58 ± 0.42	1.28 ± 0.35	0.002
TOC (mmol/L)	0.219 ± 0.079	0.209 ± 0.115	0.678
OSI	0.144 (0.094–0.193)	0.126 (0.101–0.213)	0.706
GSH (µmol/L)	17.4 (13.4–24.7)	10.6 (7.8–14.2)	<0.001
GSSG (µmol/L)	6.60 (5.38–8.75)	5.73 (4.68–8.18)	0.054
GSH/GSSG	2.61 (2.26–3.10)	1.69 (1.46–2.00)	0.001

The results are presented as means ± standard deviations for normally distributed data, or medians with interquartile ranges (1Q-3Q) for non-normally distributed variables; GSH—reduced glutathione, GSSG—oxidized glutathione, TOC—total oxidant capacity, TAC—total antioxidant capacity, OSI—oxidative stress index (TOC/TAC), BALP—bone alkaline phosphatase.

**Table 3:** Differences in biochemical parameters between survivors with osteosarcoma and Ewing’s sarcoma.

	Survivors with Osteosarcoma *n* = 24	Survivors with Ewing’s Sarcoma *n* = 11	*p*
Age (years)	15.3 ± 2.9	16.5 ± 3.7	0.389
Post-therapy period (months)	14.0 (5.8–17.0)	16.0 (9.5–18.0)	0.444
BALP (U/L)	82.5 (45.3–127.1)	51.6 (36.1–110.0)	0.338
TAC (mmol/L)	1.66 ± 0.42	1.42 ± 0.41	0.120
TOC (mmol/L)	0.195 ± 0.078	0.271 ± 0.057	0.006
OSI	0.127 (0.074–0.163)	0.188 (0.165–0.214)	0.008
GSH (µmol/L)	19.2 (14.1–34.3)	14.1 (11.3–18.3)	0.092
GSSG (µmol/L)	6.48 (5.23–9.36)	6.60 (5.97–7.53)	0.826
GSH/GSSG	2.93 (2.56–3.19)	2.41 (1.61–2.70)	0.029

The results are presented as means ± standard deviations for normally distributed data, or medians with interquartile ranges (1Q-3Q) for non-normally distributed variables; GSH—reduced glutathione, GSSG—oxidized glutathione, TOC—total oxidant capacity, TAC—total antioxidant capacity, OSI—oxidative stress index (TOC/TAC), BALP—bone alkaline phosphatase.

**Table 4:** Correlations between serum concentrations of total oxidant and antioxidant capacity and glutathione concentrations in healthy children.

	TOC (mmol/L) r(*p*)	TAC (mmol/L) r(*p*)	OSI r(*p*)
GSH (µmol/L)	−0.197 (0.279)	0.394 (0.026)	−0.323 (0.072)
GSSG (µmol/L)	−0.200 (0.274)	0.068 (0.713)	−0.190 (0.299)
GSH/GSSG	−0.014 (0.940)	0.504 (0.003)	−0.209 (0.252)

GSH—reduced glutathione, GSSG—oxidized glutathione, TOC—total oxidant capacity, TAC—total antioxidant capacity, OSI—oxidative stress index (TOC/TAC), r(*p*)—Spearman’s rank correlation coefficient (*p*-value).

**Table 5:** Correlations between serum concentrations of total oxidant and antioxidant capacity and glutathione concentrations in bone tumor survivors.

	TOC (mmol/L) r(*p*)	TAC (mmol/L) r(*p*)	OSI r(*p*)
GSH (µmol/L)	−0.132 (0.449)	0.384 (0.023)	−0.329 (0.054)
GSSG (µmol/L)	−0.104 (0.554)	0.413 (0.014)	−0.263 (0.127)
GSH/GSSG	−0.087 (0.619)	−0.002 (0.992)	−0.175 (0.315)

GSH—reduced glutathione, GSSG—oxidized glutathione, TOC—total oxidant capacity, TAC—total antioxidant capacity, OSI—oxidative stress index (TOC/TAC), r(*p*)—Spearman’s rank correlation coefficient (*p*-value).

## Data Availability

Data is contained within the article.
